# Racial and Rural Disparities in Stroke Discharge Disposition Stratified by 90-Day Survival Status

**DOI:** 10.1016/j.jamda.2025.105847

**Published:** 2025-09-09

**Authors:** Mengyuan Cheng, Winston Kennedy, Brady Post, Nasim Ferdows

**Affiliations:** a Department of Public Health and Health Sciences, Bouvé College of Health Sciences, Northeastern University, Boston, MA, USA; b Department of Physical Therapy, Human Movement, and Rehabilitation Sciences, Bouvé College of Health Sciences, Northeastern University, Boston, MA, USA; c School of Public Policy and Urban Affairs, College of Social Sciences and Humanities, Northeastern University, Boston, MA, USA

**Keywords:** Stroke, discharge disposition, racial disparities, rural health, Medicare, post-acute care

## Abstract

**Objectives:**

To examine racial and rural-urban disparities in discharge disposition following hospitalization for ischemic stroke, stratified by 90-day postdischarge survival status.

**Design:**

Retrospective cross-sectional study using Medicare claims data.

**Setting and Participants:**

A nationally representative 5% sample of Medicare Fee-For-Service beneficiaries hospitalized with a primary diagnosis of ischemic stroke in 2021 (N = 6804); 18% identified as racial or ethnic minorities, and 19% resided in rural areas.

**Methods:**

Data were drawn from the 2021 Inpatient Standard Analytical Files and Medicare Beneficiary Summary File. The primary outcome was discharge disposition, categorized as (1) home or home health care, (2) skilled nursing facility (SNF) or inpatient rehabilitation facility (IRF), or (3) hospice or other settings. Race or ethnicity and rurality were key independent variables, with rurality categorized using Rural-Urban Commuting Area (RUCA) codes. We stratified analyses by survival status (died during hospitalization or within 90 days vs survived beyond 90 days). Multinomial logistic regression models adjusted for demographic, clinical, and socioeconomic covariates.

**Results:**

Among all beneficiaries, 44.4% were discharged to home or home health care, 39.9% to SNF or IRF, 10.5% to hospice or another hospital, and 5.2% died during hospitalization. Among those who survived to discharge, 13.6% died within 90 days. Among these, Black and Hispanic patients had lower odds of discharge to hospice or other settings compared with White patients [odds ratio (OR) 0.38, 95% CI 0.20–0.73]. Patients residing in rural-adjacent areas had lower odds of discharge to hospice or other settings compared with urban counterparts (OR 0.56, 95% CI 0.31–1.01). No significant disparities were found among survivors.

**Conclusions and Implications:**

Disparities in discharge disposition were concentrated among patients nearing end of life. These findings highlight gaps in hospice and institutional care access for rural and minority populations, underscoring the need for targeted policy and clinical interventions to promote equity in poststroke care transitions.

Stroke is a leading cause of long-term disability and mortality in the United States and globally. According to the 2024 American Heart Association update, stroke remains one of the most burdensome diseases worldwide, contributing significantly to years lived with disability and health care system costs.^1,2^ Recovery from ischemic stroke depends heavily on the care received during the post-acute period, when rehabilitation services and discharge decisions play critical roles in shaping outcomes.^3,4^

Decisions regarding discharge disposition—whether to home, home with health services, a skilled nursing facility (SNF), an inpatient rehabilitation facility (IRF), or hospice—is a pivotal event in the stroke care trajectory. Prior literature has shown that discharge to institutional post-acute care is associated with higher levels of functional dependency and more severe clinical presentation, whereas discharge to home or home with health services is associated with lower severity and better prognoses.^5–7^ The decision-making around discharge destination can be influenced by stroke severity, comorbidities, hospital and provider characteristics, and availability of local resources.^8,9^

Despite these clinical drivers, disparities in discharge disposition persist and equitable access to optimal poststroke care settings remains a challenge. Racial and ethnic disparities in discharge disposition have been consistently documented, with Black and Hispanic patients often facing greater barriers to facility-based rehabilitation or hospice care. Several studies demonstrate that patients from racial and ethnic minority backgrounds are more likely to be discharged home, even when clinical factors may suggest a need for higher-intensity care.^10–12^ These disparities appear to be shaped by both individual-level factors (eg, insurance coverage, social determinants of health) and system-level elements such as facility availability or institutional bias.

Geographic variation adds another layer of complexity. Research shows that rural residents are less likely to access timely and guideline-concordant stroke care^13,14^ and may face challenges in accessing post-acute services because of facility shortages, longer travel distances, and reduced care infrastructure.^15,16^ For example, Shultis et al^17^ found significant gaps in acute stroke care capacity in rural communities across the Pacific Northwest.

Although the literature has established the presence of racial and geographic disparities in poststroke care independently, relatively little is known about how these disparities intersect, particularly among patients with poor short-term outcomes such as in-hospital death or early mortality. Furthermore, end-of-life transitions such as referral to hospice or comfort care are less frequently studied despite their critical importance for quality of care in the final days of life.^18,19^ This study builds on prior work by not only examining racial and rural-urban disparities in discharge disposition but also stratifying by survival status to capture differences in post-acute and end-of-life transitions—areas that have received limited attention in prior stroke literature.

To address these gaps, this study investigates racial and rural-urban disparities in discharge disposition among Medicare beneficiaries hospitalized for ischemic stroke, stratified by 90-day postdischarge survival. We focus on whether these disparities differ based on postdischarge survival status—specifically comparing patients who died during hospitalization or within 90 days postdischarge with those who survived longer. These findings have important implications for designing equitable stroke recovery pathways and improving hospice and rehabilitation access for underserved populations. Because our analysis is based on Medicare data, findings should be interpreted within the context of the US health care system and its post-acute care infrastructure.

## Methods

### Study Design and Data Source

This retrospective cohort study used 2021 Medicare data from the Inpatient Standard Analytical Files and the Medicare Beneficiary Summary File (MBSF). These data sets provide comprehensive demographic, clinical, and utilization data on Medicare beneficiaries. The study population was drawn from a 5% Medicare Standard Analytic Files, which represent a nationally representative random sample of Medicare Fee-For-Service beneficiaries selected using Medicare Health Insurance Claim (HIC) numbers.

We identified beneficiaries hospitalized with a primary diagnosis of ischemic stroke using the *International Classification of Diseases, Tenth Revision, Clinical Modification* (*ICD-10-CM*), code I63. Patients were included if they had a hospitalization for ischemic stroke in 2021 and were continuously enrolled in Medicare Parts A and B during the entire calendar year. Individuals with missing data on stroke severity, assessed by the National Institutes of Health Stroke Scale (NIHSS), were excluded. The final analytic sample included 6804 beneficiaries. We focused on 2021, the most recent year of Medicare data available at the time of analysis. Although this period overlaps with the COVID-19 pandemic, our study was not designed to isolate pandemic-related effects.

### Outcome Measures

The primary outcome was discharge disposition, categorized into 3 mutually exclusive groups: (1) discharge to home or home with health care services (HH), (2) discharge to a skilled nursing facility (SNF) or inpatient rehabilitation facility (IRF), or (3) discharge to hospice, transfer to another hospital, or other settings. We also stratified the analysis based on 90-day postdischarge survival status. Patients were categorized into 2 survival groups: (1) those who died during hospitalization or within 90 days postdischarge or (2) those who survived beyond 90 days.

### Covariates

The analysis adjusted for multiple patient-level covariates. Demographic variables included age (continuous), sex (male or female), race or ethnicity (non-Hispanic White, non-Hispanic Black, Hispanic, Other). Clinical covariates included stroke severity (measured using the NIH Stroke Scale), comorbidity burden (based on the Charlson Comorbidity Index), and diagnosis of depression (yes or no). Socioeconomic variables included Medicare-Medicaid dual eligibility (yes or no) and residence in a Medicaid expansion state (yes or no). Hospital-related covariates included length of stay (continuous), measured as the number of days in the inpatient hospital stay for the index stroke admission, and an interaction term between stroke severity and length of stay. Rurality was classified using Rural-Urban Commuting Area (RUCA) codes into 3 levels: urban, rural-adjacent, and rural-nonadjacent.^20^

### Statistical Analysis

We used multinomial logistic regression models to examine the association between patient characteristics and discharge disposition, treating discharge to home or home health care as the reference category. The model adjusted for all covariates listed above. To explore whether observed disparities varied by survival outcomes, all analyses were stratified into 2 groups: (1) beneficiaries who died during hospitalization or within 90 days postdischarge and (2) those who survived beyond 90 days.

We report adjusted odds ratios (ORs) and 95% CIs comparing the odds of being discharged to SNF or IRF or to hospice or other settings, relative to discharge to home or home health care. A detailed model specification, including all parameter definitions and the regression equation, is provided in Supplementary Material 1.

All statistical analyses were performed using R version 4.2.1. A 2-tailed *P*-value <.05 was considered statistically significant.

## Results

### Study Population

We identified 49,857 Medicare beneficiaries with a diagnosis code indicating stroke in 2021. Among these, 12,613 had an inpatient hospitalization, and 5809 were excluded due to missing National Institutes of Health Stroke Scale (NIHSS) scores. The final study sample included 6804 beneficiaries hospitalized with ischemic stroke.

Among the analytic sample, 44.4% (3019) were discharged to home or home health care services, 39.9% (2714) to an inpatient rehabilitation facility (IRF) or skilled nursing facility (SNF), and 10.5% (717) to hospice or were transferred to other hospitals. In-hospital death occurred in 5.2% (n = 354) of patients. Among those discharged alive, 13.6% died within 90 days postdischarge, with death rates of 5.2% in the home or home health care group, 11.4% in the IRF or SNF group, and 57.5% in the hospice or other group.

### Patient Characteristics by Discharge Category

As shown in Table 1, patients discharged to home or home health care were younger on average (mean age, 74.5 years) compared with those discharged to IRF or SNF (77.1 years), hospice or other (78.9 years), or who died in hospital (78.9 years). The proportion of female patients was highest in the hospice group (57.6%) and lowest in the home group (50.7%). Across all discharge categories, the majority of patients were White (≥81%).

Patients discharged to home or home health care most commonly experienced minor strokes (71.1%), whereas severe strokes were most prevalent among patients discharged to hospice or other (29.8%) or who died in hospital (44.4%). Comorbidity burden also varied by disposition; severe comorbidity (Charlson score ≥6) was observed in 50.1% of the hospice group and 49.8% of the IRF or SNF group. Depression was most frequently observed in the IRF or SNF group (44.4%) and least common among those who died in hospital (15.0%). Patients discharged to IRF or SNF also had longer median lengths of stay (6.0 days, interquartile range 4.0–9.0) compared to those discharged to home (3.0 days, interquartile range 2.0–5.0).

### Multinomial Regression Analysis

**Fig. 1.**
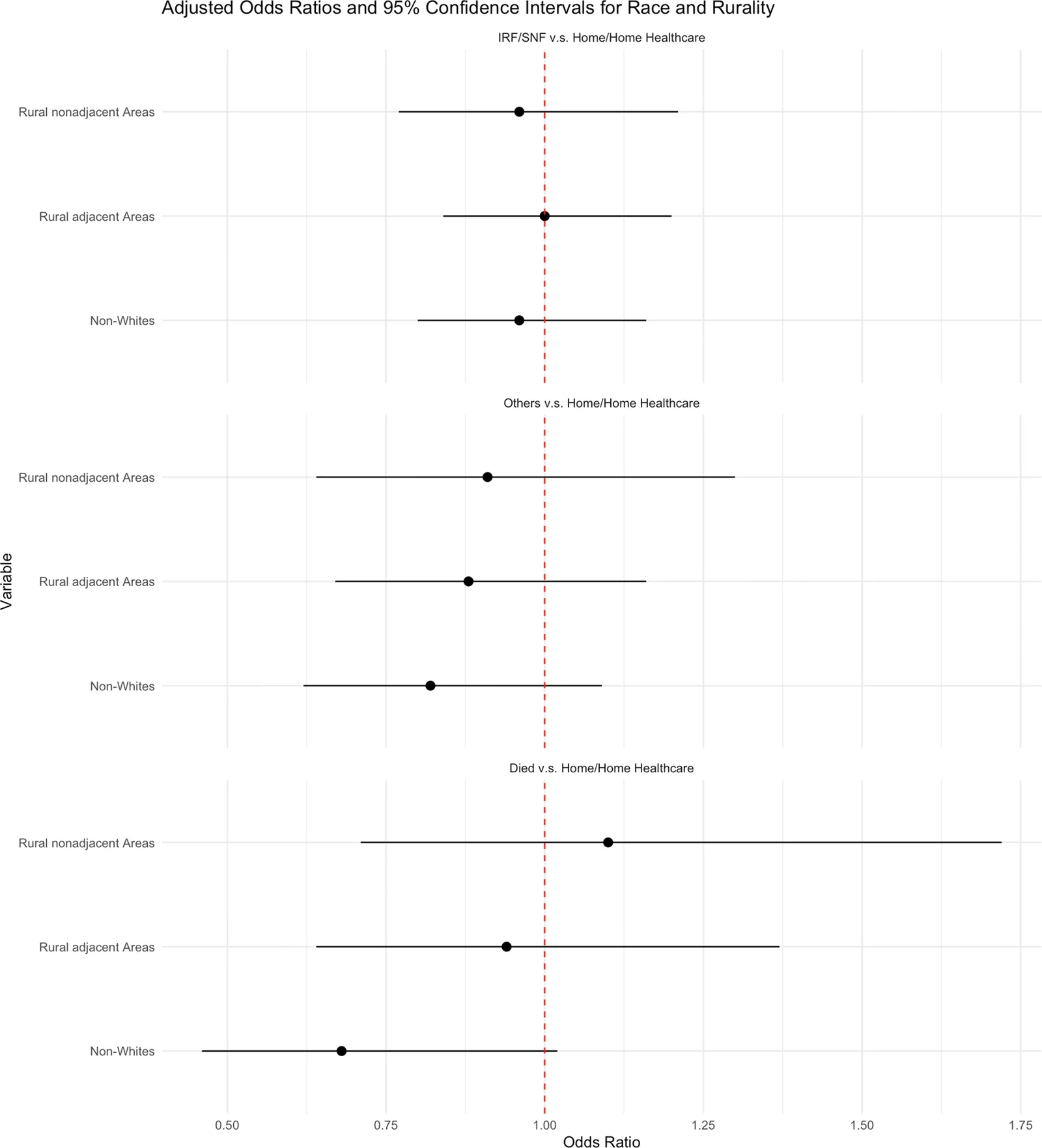
Adjusted ORs for discharge disposition among all stroke patients. Adjusted ORs and 95% CIs for rural residence and race or ethnicity comparing 3 discharge outcomes—IRF or SNF, hospice or other transfer, and in-hospital death—relative to discharge to home or home health care. ORs are adjusted for age, sex, race, stroke severity, comorbidity burden (Charlson Comorbidity Index), depression, length of stay, dual-eligibility status, Medicaid expansion state, and an interaction between stroke severity and length of stay.

Figure 1 presents the results of the multinomial logistic regression for the full sample. Higher stroke severity and comorbidity burden were associated with increased odds of discharge to IRF or SNF and hospice or other settings compared with home or home health care. Several covariates including age, depression, and dual eligibility status were also associated with discharge disposition.

### Disparities by Race and Rurality

**Fig. 2.**
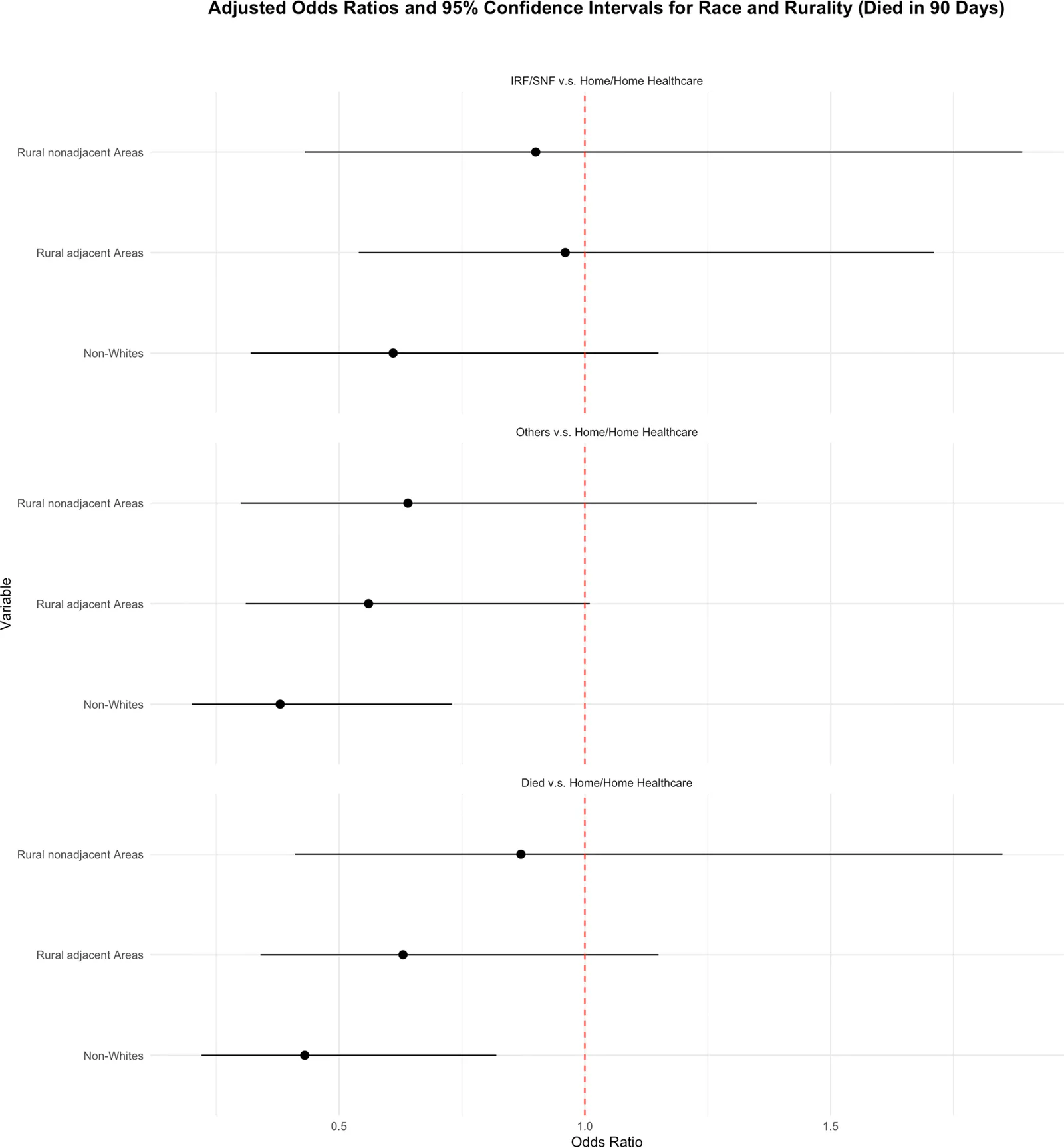
Adjusted ORs for discharge disposition among patients who died in-hospital or within 90 days postdischarge. Multivariable-adjusted ORs and 95% CIs for rural residence and race or ethnicity comparing discharge outcomes (IRF or SNF, hospice or other, and in-hospital death) relative to home or home health care for stroke patients who died during hospitalization or within 90 days postdischarge. Covariates adjusted for as in Figure 1.

As shown in Figure 2, among patients who died during hospitalization or within 90 days postdischarge, racial and rural-urban disparities were evident. Compared with non-Hispanic White patients, Black and Hispanic patients had significantly lower odds of discharge to hospice or other institutional care settings (OR 0.38, 95% CI 0.20–0.73). Patients classified as other race or ethnicity had similarly lower odds (OR 0.26, 95% CI 0.11–0.62). Patients residing in rural-adjacent areas had lower odds of discharge to hospice or other settings compared with urban residents (OR 0.56, 95% CI 0.31–1.01), whereas a similar trend was observed for rural-nonadjacent areas (OR 0.64, 95% CI 0.33–1.25), although not statistically significant.

**Fig. 3.**
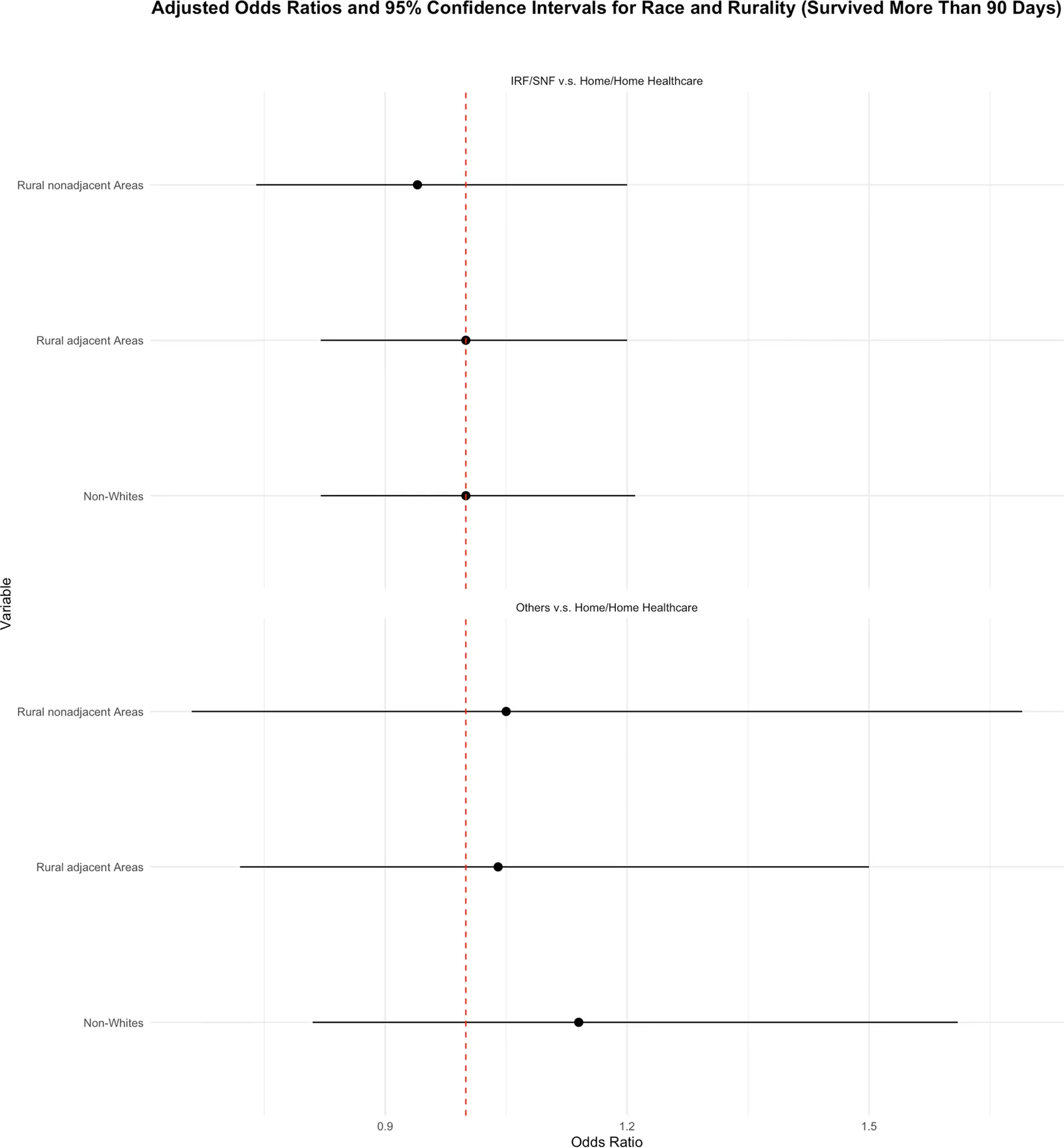
Adjusted ORs for discharge disposition among patients who survived more than 90 days postdischarge. Adjusted ORs and 95% CIs comparing discharge disposition among stroke patients who survived beyond 90 days postdischarge. Covariates include demographics, clinical characteristics, dual eligibility, depression, Medicaid expansion, and hospital-related factors, including interaction between stroke severity and length of stay.

In contrast, Figure 3 displays findings from patients who survived beyond 90 days postdischarge. In this subgroup, no statistically significant associations were found between discharge disposition and either race or ethnicity or rurality, although point estimates continued to suggest modest disparities in discharge to SNF or IRF among rural residents.

Together, Table 1 and Figure 1 through 3 illustrate both clinical predictors and disparities in discharge disposition, reinforcing the significance of patient characteristics, stroke severity, and social factors in shaping post-acute stroke care trajectories.

## Discussion

This study introduces a novel approach by stratifying discharge disposition outcomes by 90-day postdischarge survival status. While many studies examine disparities in poststroke care, few distinguish between those who survive long-term and those who die shortly after discharge. This approach allowed us to identify disparities—particularly in transitions to hospice and end-of-life care—that may be masked in aggregate analyses. In this nationally representative sample of Medicare beneficiaries hospitalized for ischemic stroke, we observed significant disparities in discharge disposition by race or ethnicity and rurality, especially among patients who died during hospitalization or within 90 days postdischarge. These findings reinforce concerns about structural inequities in access to hospice and high-intensity post-acute care, even after accounting for stroke severity and comorbidity burden.

Our results align with prior research showing that racial and ethnic minority patients are less likely to receive institutional post-acute care. For example, Lee and colleagues^12^ found that Hispanic patients had lower odds of institutional post-acute care placement compared with White patients, especially when accounting for Medicaid status. Similarly, Man and colleagues^21^ reported significant insurance-dependent racial and ethnic disparities in post-acute service use, with lower odds of facility or home health discharge among uninsured Hispanic patients. These findings suggest that differences in discharge disposition reflect not only clinical need but also insurance status and broader social determinants of health.

Geographic disparities were also evident in our analysis. Patients from rural-adjacent areas had lower odds of discharge to hospice or transfer to another institutional setting, a finding aligned with prior work by Morrow and colleagues,^15^ who documented reduced access to rehabilitation services in rural and socially disadvantaged areas. This may be explained in part by supply-side limitations in rural settings, such as reduced availability of hospice facilities, skilled nursing beds, or inpatient rehabilitation programs.^10,16,21,22^ Dwyer and colleagues^14^ also observed that rural patients had fewer opportunities for guideline-concordant stroke care, which could contribute to differential discharge patterns.

Although stroke severity and comorbidity burden were the strongest predictors of discharge to institutional care, disparities persisted even after adjusting for these clinical factors. Prior literature suggests that facility and provider characteristics, as well as systemic bias or inconsistent application of discharge planning protocols, may contribute to these inequities.^6,13^ Additionally, discharge disposition may be influenced by implicit provider perceptions of social support, caregiver availability, or anticipated follow-up compliance, factors that may correlate with race, ethnicity, or rurality.^11,23–25^

The lack of significant disparities among stroke survivors who lived beyond 90 days may reflect the diminished role of provider decision making in the longer postdischarge trajectory. However, the finding that minority and rural patients nearing end of life were less likely to be discharged to hospice or similar settings raises concern about unmet needs for palliative care or potential gaps in communication and care planning. Leifheit and colleagues^26^ found that dual-eligible patients had higher long-term mortality and readmission rates after stroke, highlighting the importance of effective and equitable transitions of care in this population.

### Limitations

This study has several limitations. First, the cross-sectional design precludes causal inference. Second, although we adjusted for a broad set of covariates, we lacked direct measures of caregiver availability, social or family support—factors that can strongly influence discharge disposition—as well as hospital-level factors. Third, stroke severity was captured using the NIH Stroke Scale, which has demonstrated reliability and predictive value^5,27^ but may not fully capture all dimensions of functional status or cognitive impairment. Additionally, because our analysis focused on data from 2021, which overlaps with the COVID-19 pandemic, some observed disparities may reflect pandemic-related disruptions in care. However, isolating the effects of COVID-19 was outside the scope of this study.

## Conclusions and Implications

Our findings highlight persistent racial and geographic disparities in poststroke discharge disposition—disparities that were most pronounced among patients with limited survival. Even after adjusting for stroke severity, comorbidity, and socioeconomic status, Black, Hispanic, and rural-residing patients were less likely to be discharged to hospice or institutional care when nearing the end of life. This suggests potential barriers in accessing appropriate post-acute or palliative care and reflects broader structural inequities in the health care system.

These patterns raise critical equity concerns and highlight the need for system-level interventions. Targeted efforts such as expanding hospice and post-acute services in rural communities, implementing culturally responsive discharge planning protocols, and promoting transparency in discharge decision-making could help mitigate disparities in stroke recovery pathways. Incorporating equity metrics into hospital discharge practices may also support more equitable transitions of care for minoritized and underserved populations. Future research should explore how policy and organizational factors influence these disparities and test interventions designed to promote fairness in end-of-life stroke care.

## Supplementary Material

SM1

SM2

Supplementary Data

Supplementary data related to this article can be found online at https://doi.org/10.1016/j.jamda.2025.105847.

## Figures and Tables

**Table 1:** Baseline Characteristics of Medicare Beneficiaries Admitted to Acute Hospital With Ischemic Stroke by Discharge Disposition, 2021

Variables	Home or Home Health Care (n = 3019)	IRF or SNF (n = 2714)	Hospice and Other Hospitals (n = 717)	In-Hospital Death (n = 354)	*P* Value

n (%) died within 90 d after discharge	156 (5.2)	309 (11.4)	412 (57.5)	NA	
Age, mean (SD)	74.46 (9.87)	77.10 (10.17)	78.85 (11.87)	78.92 (10.77)	<.001
Age group, n (%)					<.001
<65	336 (11.1)	223 (8.2)	65 (9.1)	27 (7.6)	
65–79	1764 (58.4)	1355 (49.9)	285 (39.7)	154 (43.5)	
≥80	919 (30.4)	1136 (41.9)	367 (51.2)	173 (48.9)	
Female, n (%)	1531 (50.7)	1472 (54.2)	413 (57.6)	201 (56.8)	.001
Race, n (%)					.55
White	2463 (81.6)	2200 (81.1)	586 (81.7)	288 (81.4)	
Non-White (Black and Hispanics)	386 (12.8)	369 (13.6)	97 (13.5)	40 (11.3)	
Others	170 (5.6)	145 (5.3)	34 (4.7)	26 (7.3)	
Urban-rural classification, n (%)*					.79
Urban	2434 (80.8)	2171 (80.1)	581 (81.0)	277 (78.5)	
Rural adjacent	377 (12.5)	345 (12.7)	86 (12.0)	44 (12.5)	
Rural nonadjacent	202 (6.7)	194 (7.2)	50 (7.0)	32 (9.1)	
NIHSS, n (%)					<.001
Minor stroke	2148 (71.1)	1068 (39.4)	187 (26.1)	30 (8.5)	
Moderate stroke	722 (23.9)	1228 (45.2)	207 (28.9)	94 (26.6)	
Moderate to severe stroke	68 (2.3)	217 (8.0)	109 (15.2)	73 (20.6)	
Severe stroke	81 (2.7)	201 (7.4)	214 (29.8)	157 (44.4)	
Charlson Comorbidity Index Score, n (%)					<.001
Mild (score 1–2)	546 (18.1)	195 (7.2)	51 (7.1)	30 (8.5)	
Moderate (score 3–4)	951 (31.5)	715 (26.3)	193 (26.9)	116 (32.8)	
Moderate to severe (score 5)	420 (13.9)	452 (16.7)	114 (15.9)	44 (12.4)	
Severe (score 6–21)	1102 (36.5)	1352 (49.8)	359 (50.1)	164 (46.3)	
Depression, n (%)	741 (24.5)	1206 (44.4)	211 (29.4)	53 (15.0)	<.001
Length of stay, median (IQR)	3.00 (2.00, 5.00)	6.00 (4.00, 9.00)	5.00 (3.00, 9.00)	6.00 (3.00, 10.00)	<.001
Had PT evaluation in 90 days, n (%)	552 (18.3)	464 (17.1)	54 (7.5)	3 (0.8)	<.001
Inpatient encounter count, median (IQR)	1.00 (1.00, 2.00)	2.00 (2.00, 3.00)	2.00 (1.00, 3.00)	1.00 (1.00, 1.00)	<.001
Outpatient encounter count, median (IQR)	5.00 (1.00, 11.00)	5.00 (2.00, 10.00)	2.00 (0.00, 7.00)	2.00 (0.00, 4.00)	<.001
Medicare-Medicaid dual eligibility, n (%)					<.001
Dual eligible	491 (16.3)	577 (21.3)	165 (23.0)	71 (20.1)	
Non—dual eligible	2528 (83.7)	2137 (78.7)	552 (77.0)	283 (79.9)	
Medicaid expansion State, n (%)					.013
Yes	2056 (68.1)	1816 (66.9)	470 (65.6)	268 (75.7)	
No	955 (31.6)	894 (32.9)	247 (34.4)	85 (24.0)	
NA	8 (0.3)	4 (0.1)	0 (0.0)	1 (0.3)	

IQR, interquartile range; NA, not applicable; NIHSS, National Institutes of Health Stroke Scale; PT, physical therapy.

*P* values reflect results from χ^2^ tests for categorical variables and Kruskal-Wallis tests for continuous variables.

* 6 of 3019, 4 of 2714, and 1 of 354 missing.
