# Supplementary material for: Racial and Rural Disparities in Stroke Discharge Disposition Stratified by 90-Day Survival Status
Source: J Am Med Dir Assoc. Author manuscript; Available in PMC 2026 Sep 14. (PMC13573657; doi:10.1016/j.jamda.2025.105847)
Supplement: Supplementary file 1 [file NIHMS2204895-supplement-SM1.docx]

**Methods Supplement**

***Multinomial Logistic Regression Model Specification***

The following multinomial logistic regression model was used to estimate the association between patient characteristics and discharge disposition after hospitalization for ischemic stroke. The dependent variable was discharge disposition, with three categories:

- Home or home with healthcare services (reference group)
- Skilled Nursing Facility (SNF) or Inpatient Rehabilitation Facility (IRF)
- Hospice, transfer to another hospital, or other settings

The model was specified as follows:

log(P(Yᵢ = j) / P(Yᵢ = Home)) = β₀ⱼ + β₁ⱼ·Ageᵢ + β₂ⱼ·Sexᵢ + β₃ⱼ·Race/Ethnicityᵢ + β₄ⱼ·StrokeSeverityᵢ + β₅ⱼ·Comorbidityᵢ + β₆ⱼ·LengthOfStayᵢ + β₇ⱼ·DualEligibleᵢ + β₈ⱼ·MedicaidExpansionᵢ + β₉ⱼ·Depressionᵢ + β₁₀ⱼ·(StrokeSeverityᵢ × LengthOfStayᵢ) + εᵢ

Where:

- Yᵢ: Discharge disposition for patient i
- j: Outcome category (SNF/IRF or Hospice/Other)
- Ageᵢ: Age in years
- Sexᵢ: Male or Female
- Race/Ethnicityᵢ: Categorical variable (non-Hispanic White, non-Hispanic Black, Hispanic, Other)
- StrokeSeverityᵢ: NIH Stroke Scale score
- Comorbidityᵢ: Charlson Comorbidity Index score
- LengthOfStayᵢ: Number of days hospitalized
- DualEligibleᵢ: Medicare-Medicaid dual eligibility (yes/no)
- MedicaidExpansionᵢ: Residency in Medicaid expansion state (yes/no)
- Depressionᵢ: Diagnosis of depression (yes/no)

Separate models were estimated for patients who died during the hospitalization or within 90 days post-discharge and for those who survived beyond 90 days.
